# Architectural design of anode materials for superior alkali-ion (Li/Na/K) batteries storage

**DOI:** 10.1038/s41598-024-54214-6

**Published:** 2024-02-17

**Authors:** Afsaneh Ghahari, Heidar Raissi

**Affiliations:** Department of Chemistry, University of Birjand, Birjand, Iran

**Keywords:** LIBs, SIBs, PIBs, Molecular dynamic simulation, LAMMPS, Environmental sciences, Solid Earth sciences, Chemistry, Energy science and technology, Engineering, Materials science, Nanoscience and technology, Physics

## Abstract

Developing high-performance anode materials remains a significant challenge for clean energy storage systems. Herein, we investigated the (MXene/MoSe_2_@C) heterostructure hybrid nanostructure as a superior anode material for application in lithium, sodium, and potassium ion batteries (LIBs, SIBs, and PIBs). Moreover, the anode structure’s stability was examined via the open-source Large-scale atomic/molecular massively Parallel Simulator code. Our results indicated that the migration of SIBs toward the anode material is significantly greater than other ions during charge and discharge cycles. Therefore, SIBs systems can be competitive with PIBs and LIBs systems. In addition, the average values of the potential energies for the anode materials/ions complexes are about ~ − 713.65, ~  − 2030.41, and ~ − 912.36 kcal mol^−1^ in systems LIBs, SIBs, and PIBs, respectively. This study provides a rational design strategy to develop high-performance anode materials in SIBs/PIBs/LIBs systems, which can be developed for other transition metal chalcogenide-based composites as a superior anode of alkali metal ion battery storage systems.

## Introduction

The development of sustainable and clean energy sources is one of the most pressing issues in today’s society as an alternative to fossil fuel technologies^1,2^. Notwithstanding these advances, rechargeable battery emerges as the optimal choice for an energy storage system due to their minimal maintenance needs, high efficiency, and extended cycle life in energy conversion^3,4^. In the construction of a smart-grid energy storage system (ESS), the development of secondary batteries necessitates larger quantities of materials^5,6^. As a result, striking a delicate balance among crucial factors, such as power density, life, energy density, safety, and cost, becomes imperative for these secondary batteries^7–10^. Alkali metal ion batteries, such as lithium-ion batteries (LIBs), sodium-ion batteries (SIBs), and potassium-ion batteries (PIBs), are essential rechargeable battery technologies that are essential for renewable energy applications^6,11–13^. For instance, the LIBs rechargeable battery has dominated the electric vehicles and portable electronics market over the past two decades^14–16^. However, the uneven distribution and insufficient Lithium resources raised some serious concerns about the future deployment of LIBs^17,18^. Over the last few decades, there has been significant progress in exploring battery alternatives beyond LIBs. Among these, sodium and potassium-ion batteries (SIBs/PIBs) are gaining increasing attention due to their promising potential. Next-generation batteries based on Na^+^ and K^+^, alkali ions common in the earth’s crust, are considered promising alternatives in future energy storage due to their abundance, high energy density and low cost^19–24^. Meanwhile, non-aqueous SIBs and PIBs have also been developed during the last decades, inspired by the lack of Li^+^ resources^25,26^. Whereas compared with that of Li^+^ (0.76 Å, and molar mass 6.94 g mol^−1^), the larger ionic radius (Na^+^ = 1.02 Å, and K^+^ = 1.38 Å) and the heavier atomic weights (Na^+^ = 22.99 g mol^−1^, K^+^ = 39.10 g mol^−1^) of Na^+^/K^+^ lead to lots of trouble including higher diffusion barriers^27–29^. On the other hand, the larger ionic radius causes sluggish kinetics^30,31^, large volume change during insertion/extraction processes within the existing electrode materials^32,33^, poor cycling stability^34–37^, and short cycle life^38–40^. Hence, identifying an appropriate electrode material capable of hosting various alkali ions poses a significant challenge due to the expanding ionic radius and distinct interactions with the host electrodes^41,42^. Hence, it is urgent to design advanced electrode materials with robust structures that allow fast diffusivity and high reversibility of Na^+^ and K^+^, realizing rate capability, high capacity and outstanding cycling stability^41–49^. Graphite is the most widely used commercial anode material for LIBs, owing to increased battery life, energy storage, and fast charging capability^49–55^, and it has attracted much attention from scientists. In addition, graphite is unsuitable for high-energy-density AMIBs due to its small spacing interlayer and does not provide meaningful capacity, particularly for SIBs/PIBs^56,57^. Meanwhile, SIBs exhibit chemical behaviors similar to LIBs; however, the substantial ionic radius of Na^+^ (1.02 Å) inevitably leads to significant volume expansion during charge/discharge processes. Consequently, SIBs frequently exhibit restricted capacity and cycling instability when compared to LIBs. Hence, the central focus for enhancing the electrochemical performance of SIBs lies in the advancement of electrode materials characterized by both high capacity and prolonged cycle life. The exploration of two-dimensional (2D) materials in rechargeable batteries has received great attention as promising anode materials owing to, the capability of higher charging rates and their high surface area by metal ions^58,59^. Therefore, developing anode materials with excellent comprehensive electrochemical performance and low cost is crucial for the wide-scale practical application of SIBs/PIBs^60^. MXenes, also recognized as 2D transition metal carbides, are becoming increasingly promising for the storage of sodium ions. This is due to their outstanding metallic electronic conductivity, which can reach up to 9880 S cm^−1^, along with a low diffusion barrier for Na^+^ falling within the range of 0.1–0.2 eV^61,62^.

In recent years, there has been a rapid increase in research interest in carbon anodes in SIBs and PIBs, driven by the comparable electrochemical advantages of metallic Na and K compared to Li. Additionally, the reserves of sodium and potassium metals are positioned 6th (constituting 2.36% by weight) and seventh (making up 2.09% by weight)^63^, respectively. These resources far exceed those of Li, which comprises only 0.0017% in weight^63^. Up to date, metal chalcogenides, such as selenides, metal oxides, and sulfides, have been extensively studied as promising active electrode composites for SIBs and PIBs. These materials are favored for their abundance in the Earth’s crust and high specific capacity, making them attractive candidates for advancing battery technologies. Amongst various anode materials, metal selenides, known for their cost-effectiveness, resource abundance, and relatively stable electrochemical performance, have garnered increasing attention. Over the past years, have attracted much attention for interest in insertion-type anodes, including Ti-based and carbonaceous, for advanced AMIB due to their stable cycling performance. Regarding these materials, they frequently demonstrate relatively theoretical capacities, typically below 280 mA h g^−1^. Zhao et al.^64^ developed the creation of 2D MoSe_2_@graphene nanocomposites tailored for Na^+^ capacitors, achieving an impressive energy density of 82 Wh kg^−1^ with a corresponding power density of 63 W kg^−1^. Despite their potential, these electrodes frequently face significant challenges, including substantial changes in volume during processes governing cycling. This results in the collapse of the active material’s structure, which leads to rapid capacity deterioration. Therefore, creating electrode materials with robust structures for effective storage of Li^+^/Na^+^ and K^+^ with high performance remains a significant challenge. Simultaneously, additional research is needed to explore multifunctional electrode materials that offer both high capacity and extended lifespans, addressing the challenges posed by the energy crisis. Additionally, MoSe_2_ is recognized as an inherent semiconductor possessing a band gap of approximately 1.1 eV, narrower than that of MoS_2_ (around 1.7 eV). This narrower band gap suggests higher electronic conductivity, a quality advantageous for achieving a high-rate capability. These characteristics collectively make MoSe_2_ a promising candidate for the electrode anode material in SIBs. MoSe_2_ electrodes face difficulties, such as instability and inherent low electronic conductivity. These issues lead to subpar rate performance and cycling stability in practical applications. Researchers have utilized different carbon materials to generate hybrids or nanocomposites incorporating MoSe_2_ to enhance its anode material properties, addressing issues such as low electrical conductivity and structural stability^13,65^. Graphene’s exceptional characteristics, such as expansive surface area, high conductivity^66,67^, flexibility, and compatibility with other molecules, endow it with unique potential for application in supercapacitors^68,69^. Graphite materials commonly employed in commercial LIBs face limitations in accommodating Na^+^ insertion, resulting in insufficient capacity (below 35 mAh g^−1^)^70^. Consequently, there is a critical need to develop high-performance and versatile anode materials to overcome the challenges affecting not only LIBs but also SIBs and PIBs. The layered structure of graphene, along with its high potential, has led to the study of other layered inorganic materials, including layered transition metal dichalcogenides such as SnSe_2_, SnS_2_, MoSe_2_, MoS_2_, and WS_2_^37–41^. Layered transition metal dichalcogenides (TMDs) characterized by the MX_2_ formula (where M can be Mo, Ti, Nb, Hf, Zr, Sn, V, Ta, W, and X can be Te, S, Se) exhibit a morphology of graphite-like and demonstrate impressive performance. The X atoms form the outer layer in this structure, sandwiching the arrangement of metals in a hexagonally close-packed structure, represented as X–M–X. The M–X bond within each layer is covalent, while van der Waals forces govern the interaction between layers. This unique arrangement and morphology make TMDs ideal for energy conversion and storage applications, due to their distinct electronic, chemical, and physical properties^71–73^. Two-dimension semiconductors, particularly TMDs, have attracted tremendous attention as promising candidates for the next-generation energy storage devices. The attraction is owing to the better high conductivity, surface area (that provides storage surface sites), exceptional structural stability, and layered structure of TMDs^74^. These characteristics candidate the TMDs to be more favorable contenders to store energy. It is worth mentioning that adjacent layers in TMDs materials are bounded with comparatively weak van der Waals (vdW) interactions^75^. Moreover, the uniform layered structure has provided permeable channels for inserting/extracting Na^+^ and K^+^ ions^76,77^. Recently, some theoretical studies also indicated that TMDs monolayers could substantially accelerate ion diffusion and accommodate more ions^78,79^. Molybdenum diselenide (MoSe_2_), among these TMDs, is regarded as a competitive anode material with an interlayer spacing of 6.5 Å^80^, which facilitates large-size of PIBs (1.38 Å) insertion/extraction and decreases the corresponding diffusion barrier^81,82^. Therefore, to promote the structural stability and the electrical conductivity of MoSe_2_, great efforts have been made, such as designing the hierarchical structure and heteroatomic interface engineering^83–85^. Consequently, it could effectively suppress the volume changes of MoSe_2_ and significantly increase the electron transport in the composite^86,87^. It is worth noting that the main disadvantages of MoSe_2_ materials are their electronic conductivity and low instinct density during cycling. Meanwhile, integrating the MoSe_2_ nanosheet with other low-cost conductive substrate materials is expected to increase the alkaline ion storage performance^88,89^. Therefore, one of the effective strategies is coupling molybdenum diselenide and carbon materials to improve electrical conductivity and structural stability. Transition metal selenides have attracted attention as anode materials for SIBs due to their high theoretical capacity and electronic conductivity^90,91^. Nevertheless, the pronounced volume changes associated with transition metal selenide-based anode materials result in poor cycle stability during the repetitive insertion/extraction of SIBs. The creation of a low-dimensional structure holds promise as a solution, as it not only mitigates volume expansion but also offers additional active sites for the migration of Na^+^^92^. Additionally, a common strategy to enhance the cycle stability of transition metal selenides involves their combination with other highly conductive electrodes, such as graphene, MXene, and carbon nanotubes^93^. Among the various investigated anode materials, due to its distinctive structural advantages, MoSe_2_ has garnered extensive attention as an anode material in energy storage applications^118^. MoSe_2_, with its unique 2D layered structure and high specific capacity, is a promising electrode material for SIBs. MoSe_2_ stands out among transition metal dichalcogenides (TMDCs) and similar compounds due to the significant role of Mo in conferring a metallic nature. The unique properties of the 2D layered framework, characterized by substantial layer distances, enhance the diffusion kinetics of Na^+^/K^+^. However, the relatively low electrical conductivity imposes a limitation on electron transfer, leading to poor rate capability^27–29^. Nanostructure engineering strategies are extensively used to enhance Na^+^ and K^+^ storage characteristics of molybdenum chalcogenides. In contrast to bulk materials, engineering the nanostructure reduces the ion transfer distance, enhancing ion diffusion kinetics and relieving structural stress. This improvement promotes increased reaction activity and optimal utilization of active materials. For instance, Tao et al.^94^ showed that carbon-coated MoSe_2_ nanosheets as anode for PIBs deliver a high-specific capacity of 258 mAh g^−1^ after 300 cycles at 100 mA g^−1^. Li et al.^95^ synthesized the MoSe_2_/C nano-plates sheathed in N-doped carbon (MoSe_2_/C@NC) as ideal anode material in the field of energy storage for Na^+^/K^+^. The results showed a SIBs/PIBs storage capacity of 362 and 310 mA h/g at 0.1 A/g, respectively. Moreover, Kang et al.^96^ created a core/shell nanotube structure (VSe 1.6/C@N–C⊂MoSe_2_, with VSe 1.6/C inner cores) as an anode material for Na^+^/K^+^, it exhibited markedly enhanced high cycling stability. Recently, Zhang et al. synthesized a hierarchical nanorod structure (MoSe_2_/N–C) to evaluate the practical application of MoSe_2_/N-C anode for the charge/discharge process and maintaining the structural integrity of MoSe_2_. The results showed that the MoSe_2_/N-C anode materials has a superior high-rate capability and long-cycle performance for storing Na^+^/K^+^^97^. Notwithstanding the latest improvements in MoSe_2_/C anode materials, optimizing the C/MoSe_2_ structure is still in progress and worthy of further investigation. MXenes are expressed by the general formula M_n+1_X_n_T_x_ (n = 1–3), where M is an early transition metal (such as Ti, Nb, V, etc.) X is C/N or a mixture of them, and T_x_ represents oxygen [=O], hydroxyl [–OH] and fluorine [–F]^98^. MXenes have been extensively investigated as metal electrode materials, capitalizing on their theoretically significant metal ion storage capacities and intriguing structural controllability^99^. Meanwhile, The MXene substrate is highly conductive, this contributes to the effective reduction of MoSe_2_ nanosheet aggregation and an increase in the conductivity of the electrode material. According to current developments for rechargeable batteries, 2D MXene materials are ideal hosts for LIBs, SIBs, and PIBs storage^100,101^. Given their numerous attractive qualities, including substantial interlayer spacing and a minimal Na+/K+ diffusion barrier, there exist considerable possibilities for developing electrodes specifically tailored for high-performance SIBs/PIBs. Besides, due to surface functional groups with a negative charge, they are used for the selective separation of ions in different sizes and charges^102–105^. For example, Wu et al. reported the MoS_2_ nanosheets on MXene stabilized via a carbon-based nanoplate (C), which displayed significant electrochemical properties as an ideal anode material for LIBs^106^. Also, Cao et al.^107^ designed the MXene@N-doped carbonaceous nanofiber structure as a novel electrode material for high-performance SIBs and PIBs. They found that this porous nanostructure promotes the transport and conduction of SIBs and PIBs and fully uses the intrinsic advantages of the 2D material. And also, Sun et al. designed the carbonaceous nanofiber structure (MXene@N-doped) as the anode for application in high-performance energy storage for SIBs and PIBs. Their results showed that this anode material can significantly enhance the capacitive storage of AMIBs and provides efficient charge transfer in the electrode^108^. Xu et al. also investigated the innovative ultrafast network electrode composed of MXene/MoSe_2_, which is synthesized via a simple hydrothermal method for energy storage devices and SIBs. The results revealed that the created cells demonstrate a high degree of reversibility capacities and excellent electrochemical performance of the hybrid materials, especially at high currents^109^. Besides, Liang et al. reported a three-dimensional hierarchical structure of MoSe_2_ with nitrogen and fluorine co-doped carbon (MoSe_2_/NFC) structure which facilitates Li^+^ insertion/extraction. Their findings demonstrated excellent rate performance and an exceptionally long cycling life, making them suitable for use in LIBs^110^. Recently, various computational studies have been examined to facilitate the rationalization of experimentally observed phenomena. For example, molecular dynamics (MD) simulations can predict how selected atoms in various LIBs/SIBs/PIBs systems will move over time based on an overall insight into the physics governing interatomic interactions. Herein, we can gain further insights into alkali ions (Li^+^/Na^+^/K^+^) transport mechanisms and the mobility of migration of Li^+^, Na^+^, and K^+^ toward the anode material with MD open-source code for the Large-scale Atomic/Molecular Massively Parallel Simulator (LAMMPS). Hence, to assess its suitability as an anode for PIBs/NIs/LIBs systems, we examined the properties of the system, including energies, the root means square deviation, the mean square displacement, and the radial distribution function. The results evidence that rationally designing the carbon-covered MXene/MoSe_2_ can dramatically improve their LIBs/SIBs/KIBs storage performances. Generally, our objective in this regard is to answer the following question: Did the interface of MoSe_2_ nanosheets, MXene flakes, and carbon layer as anodes help to promote charge transfer and improve structural durability for energy storage for LIBs/SIBs/PIBs by MD simulation code LAMMPS?

## Materials and methods

**Figure 1:**
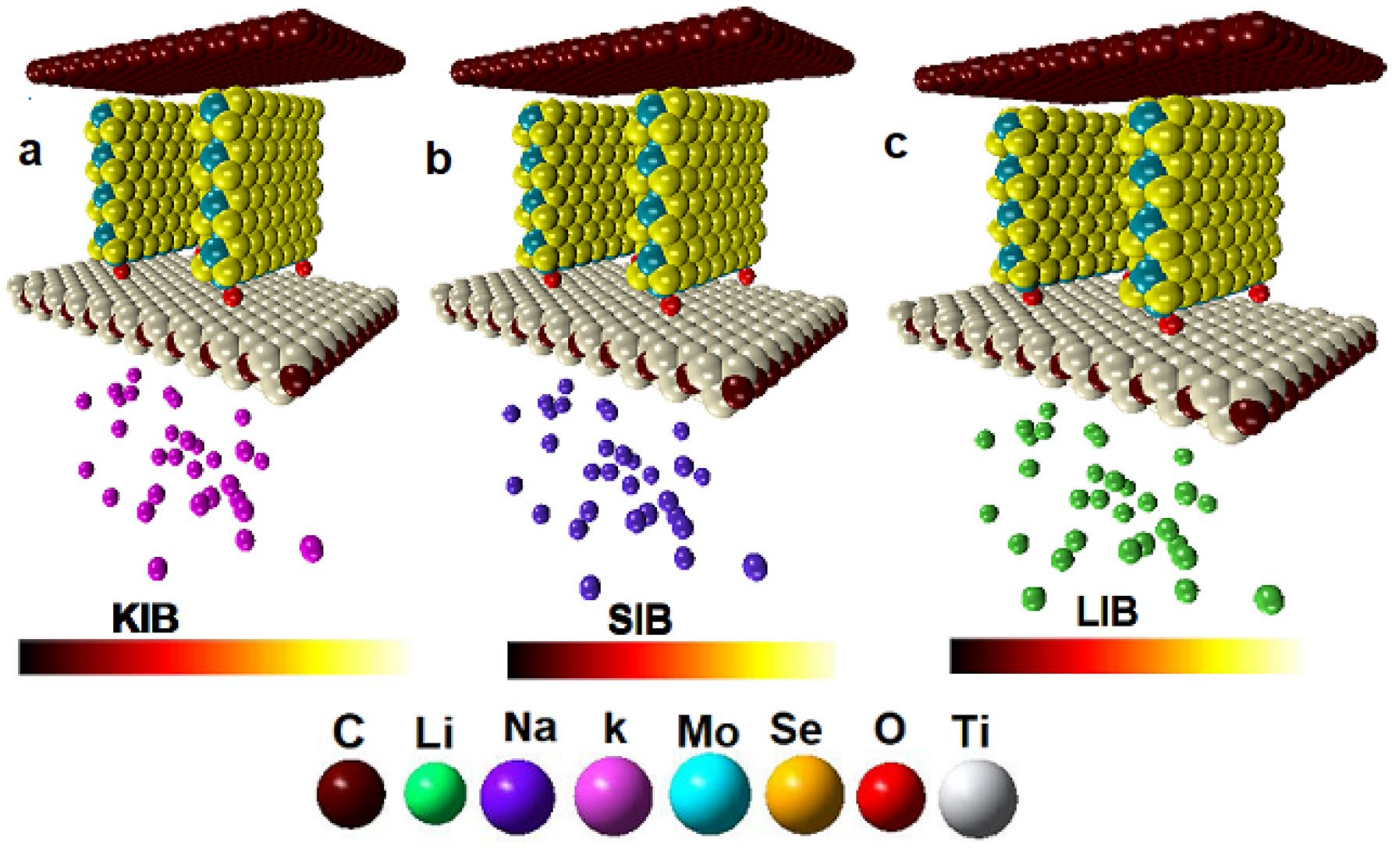
The structures of the anode materials. (**a**) PIBs system (**b**) SIBs system (**c**) LIBs system.

Herein, we investigated a well-designed structural of carbon-coated MoSe_2_ with the MXene hybrid nanosheets (MoSe_2_/MXene@C) as the anode material for LIBs/SIBs/PIBs. It is worth mentioning that the initial geometry of anode material is taken from the X-ray data by Huang et al.^111^ work. The design of the MoSe_2_/MXene@C as an anode material is illustrated in Fig. 1. In this regard, MXene nanosheets is used as a conductive substrate; thus, MoSe_2_ nanosheets are positioned vertically on the MXene surface to obtain hybrid MXene/MoSe_2_ nanosheets. It is noteworthy that the MXene flakes are anchored to the MoSe_2_ nanosheets via strong covalent bonds, which is significant for improving the hybrid structure’s stabilizing and promoting the kinetics of the migration of electrons and alkali ions throughout charge and discharge processes. In addition, the formation of Ti–O–Mo through covalent bonding at the interface between MXene and MoSe_2_ nanosheets enhances the interaction between the two components. In addition, the MoSe_2_ nanosheets have covered the surface of the MXene flake to form a 3D network of interconnected porous structures. Moreover, the carbon layer evenly covers MXene/MoSe_2_ nanocomposite, amplifying the 2D nano-structure and increasing the overall conductivity. Thus, three simulation systems are designed as promising anode materials for Li^+^, Na^+^, and K^+^ and randomly distributed around the MXene structure. Most importantly, the key point is achieving fast LIBs/SIBs/PIBs diffusion kinetics in the MoSe_2_/MXene@C interlayer. Furthermore, this strategy of the hierarchical 2D nanosheet structure of MoSe_2_/MXene@C electrode is promising for developing a high-performance with potential applications in supercapacitors and batteries.

Comprehending the alkali-metal extraction/insertion mechanism is crucial in solid-state chemistry and holds significance in utilizing it as positive electrode materials for LIBs, SIBs, and PIBs. MXenes have undergone exploration as electrode materials for storing Na^+^^112–114^. It’s noteworthy that the only work corresponding to Ti_2_CTx used as an anode electrode for SIBs was reported by Yamada and co-workers. The findings from Yin et al.^115^, Yang et al.^116^, and Li group^117^ demonstrate a complete reaction from Li_2_Se to MoSe_2_, as opposed to Se, throughout the charging process. The regeneration of MoSe_2_ serves as evidence for the reversible reaction with Li^+^, thereby confirming the reversibility outlined in Eq. (1) during the discharge/charge process.

1 $${\text{MoSe}}_{{2}} + {\text{4Li}}^{ + } + {\text{4e }} \leftrightarrow {\text{ Mo }} + {\text{ 2Li}}_{{2}} {\text{Se}}$$

The sodium storage mechanism in MoSe_2_^117^ is contingent upon the discharge potential cut-off. Specifically, when the cut-off voltage is placed higher than 0.5 V, an intercalation reaction occurs in the material, as defined by Eq. (6). Conversely, when the cut-off is set below 0.5 V, a conversion reaction takes place, as outlined in Eq. (7). Despite conversion reactions, the MoSe_2_ anode experiences an enhancement in a specific capacity; meanwhile, it results in the collapse of the layered structure in the composites, it causes the layered structure in the composites to break down, contributing to inferior cycling performance^118^. The findings lead to the conclusion that the regulation of the cut-off discharge voltage is essential for optimizing overall performance.

A summary of the reaction mechanisms (Eqs. 2, 3)^116,117^ of TMSes materials for SIBs/PIB is in the following:

2 $${\text{MaSe}}_{{\text{b}}} + {\text{xNa}}^{ + } /{\text{K}}^{ + } + {\text{ xe}}^{ - } \leftrightarrow {\text{ Na}}_{{\text{x}}} {\text{M}}_{{\text{a}}} {\text{Se}}_{{\text{b}}} \left( {{\text{K}}_{{\text{x}}} {\text{M}}_{{\text{a}}} {\text{Se}}_{{\text{b}}} } \right)$$

3 $${\text{Na}}_{{\text{x}}} {\text{M}}_{{\text{a}}} {\text{Se}}_{{\text{b}}} \left( {{\text{K}}_{{\text{x}}} {\text{M}}_{{\text{a}}} {\text{Se}}_{{\text{b}}} } \right) \, + \, \left( {{\text{2b}} - {\text{x}}} \right){\text{ Na}}^{ + } /{\text{K}}^{ + } + \, \left( {{\text{2b}} - {\text{x}}} \right){\text{ e}}^{ - } \leftrightarrow {\text{aM}} + {\text{bNa}}_{{2}} {\text{Se }}\left( {{\text{K}}_{{2}} {\text{Se}}} \right)$$

According to the Yin co-worker’s^115^ analysis, the mechanism for Na^+^ storage can be elucidated through the following reaction formulas:

4 $${\text{MoSe}}_{{2}} + {\text{4Na}}^{ + } + {\text{4e }} \to {\text{Mo }} + {\text{ 2Na}}_{{2}} {\text{S}}$$

5 $${\text{Na}}_{{2}} {\text{Se }} \leftrightarrow {\text{ Se }} + {\text{ 2Na}}^{ + } + {\text{2e}}$$

To Understanding the alkali-metal extraction/insertion mechanism^116,117^ holds significant importance not only in solid-state chemistry but also in the application of these materials utilized as electrode for LIBs, NIBs, and KIBs.

6 $${\text{MoSe}}_{{2}} + {\text{xNa}}^{ + } \leftrightarrow {\text{ Na}}_{{\text{x}}} {\text{MoSe}}_{{2}}$$

7 $${\text{Na}}_{{\text{x}}} {\text{MoSe}}_{{2}} + {4 } - {\text{ xNa}}^{ + } \leftrightarrow {\text{ Mo }} + {\text{ 2Na}}_{{2}} {\text{Se}}$$

The K storage mechanism of the MoSe_2_ in the initial cycle closely parallels that of MoSe_2_/Na. More precisely, K^+^ intercalation reactions occur at voltages exceeding 0.53 V, whereas below this threshold, conversion reactions are triggered^119^. In the following cycles, the MoSe_2_/K mechanism deviates from that of MoSe_2_/Na. The Lu group conducted ex situ XRD, Raman, and TEM analyses, revealing that the primary discharge product is K_5_Se_3_. In contrast, the charge product comprises MoSe_2_, Mo_15_Se_19_, and Se^119^.

In contrast to the pristine MoSe_2_ electrode, the MoSe_2_/MXene electrode exhibits significantly enhanced cycling performance at high rates in Na^+^ and Li^+^ half cells. This improvement can be attributed, in part, to the confining influence of the MXene.

Notably, we have investigated an innovative approach to designing Ti-based MXene@MoSe@/C electrode materials for advanced LIBs/SIBs and PIBs storage using the LAMMPS package.

The details about the simulation box size and the total number of alkali ions for each of the systems are presented in Table 1. The initial atomic coordinates are arranged using packing optimization through the Packmol program for molecular dynamics simulations^120^. Eventually, in this simulation, the periodic boundary condition (PBC) is applied along the X, Y and Z-axis, with a box dimension of 4 × 4 × 9 nm^3^. Furthermore, the periodic boundary conditions are evaluated in the x and y directions. At each time step, the forces from the MD particles that the fix acts on are applied to the ions, and the ions properties are calculated.

**Table 1:** Detail of the simulation boxes used in this study.

Systems		No. Li^+^, Na^+^, K^+^	Box size (nm^3^)
LIBs	Li-ions system	30	4 × 4 × 9
SIBs	Na-ions system	30	4 × 4 × 9
PIBs	K-ions system	30	4 × 4 × 9

For the initial structure design of the simulation, we utilized the ATOMSK program^121^. Visualization and snapshots of the simulation are obtained utilizing VMD (visual molecular dynamics)^122^ package and the graphics software package Open Visualization Tool (OVITO)^123^. All MD simulations of LIBs/SIBs/PIBs are performed using the LAMMPS program^124^ (version 2 Mar 2020), which is designed by Sandia National Laboratories (SNL)^125^, a very customizable molecular dynamics simulation software. In addition, for anode material, we need an attractive interaction to hold the components together and repulsive to prevent the overlap of the nanostructure. Therefore, the Lennard–Jones potential is often used to model this behavior in simulations^126^. The MD simulation results are strongly dependent on the choice of force field for interactions between atoms. This simulation is investigated with a Lenard Jones (LJ) force field, for anode materials. This force field quantifies energy in kcal mol^−1^, aligning with the real units. Notably, the optical atomic style for simulating batteries in the LAMMPS software is set to full. The energy potential is described through valence-bonded and nonbonded interactions.

E = E_R_ + E_θ_ + E_φ_ + E_ω_ + E_vdw_ + E_elc_ where E_R_ represents valence interactions, E_θ_ corresponds to bond angle bending, E_Φ_ denotes dihedral angle torsion, E_ω_ accounts for the inversion term, E_vdw_ covers interactions of vdW, and E_elc_ represents the electrostatic term. The following details (Eq. 8) are illustrated the nonbonded interactions (vdW forces) with potential LJ, where r_ij_ represents the distance between the interacting particles, and σ and ε determine the length scale and scale of the interaction strength, respectively.

The cut-off radius is shown with “${{\text{r}}}_{{\text{c}}}$”, set as 12–15 Å in all simulations. The parameters σ and ε depend on the type of atoms in the simulated structure^127^, In this particular model, the nonbonded interactions, specifically the van der Waals forces, through the Lennard–Jones potential are represented in the following:

The potential is defined as:

8 $${\text{U}}\left( {r_{ij} } \right) = {4}\upvarepsilon \left[ { \, \left( {{\raise0.7ex\hbox{$\sigma $} \!\mathord{\left/ {\vphantom {\sigma {r_{ij} }}}\right.\kern-0pt} \!\lower0.7ex\hbox{${r_{ij} }$}}} \right)^{12} - \left( {{\raise0.7ex\hbox{$\sigma $} \!\mathord{\left/ {\vphantom {\sigma {r_{ij} }}}\right.\kern-0pt} \!\lower0.7ex\hbox{${r_{ij} }$}}} \right)^{6} } \right]\quad {\text{if}}\,r_{ij} \le r_{c}$$

The 1/r^6^ term is related to vdW attraction, while the 1/r^12^ term is related to the repulsion between electron clouds.

For L–J interactions between different atom types, parameters ε_i,j_ and σ_i,j_ are calculated using Lorentz − Berthelot combining rules^128^:

9 $$\varepsilon_{ij} = \sqrt {\upvarepsilon _{ij} \, \upvarepsilon _{ij} }$$

10 $$\sigma_{ij} = \frac{{\sigma_{ij} + \sigma_{ij} }}{2}$$

where ε and $\upsigma$ refer to the Lennard–Jones energy and distance are applied to estimate the parameters for atom-pairs. The values Lennard–Jones parameters of the current study are presented in Table 2.

**Table 2:** Lennard–Jones Pair Potential Parameters.

Elements	σ_*i*_ (Å)	ε_*i*_ (kcal mol^−1^)
C_1_	3.166	0.7818
C	0.3563	0.46024
Mo	0.08	2.73
Li	0.43509	1.4397
Na	2.719	2.432
K	0.10	3.047
O	3.5532	0.75
Se	3.3875	2.6066
Ti	0.282	0.071

The ε and σ constants of L–J potential function in the current study.

Interatomic interactions, such as dihedral, bond strength, inversion, angle torsion, terms bond strength, and bond-angle bend for a single atom, can be computed using the equations provided below:

11 $${\text{E }} = \frac{1}{2}{\text{ K}}_{{\text{r}}} \left( {{\text{r}} - {\text{r}}_{0} } \right)$$

The harmonic oscillator constant used in the MD simulation is 300 (Kcal/mol)/A^2^ where K_r_ represents the harmonic oscillator constant and r_0_ is the atomic bond length. Moreover, the angle energies are obtained from the following equation:

12 $${\text{E }} = \frac{1}{2} {\text{K}}_{\uptheta } \left( {\uptheta -\uptheta _{0} } \right)^{{2}}$$

The oscillator’s harmonic angle constant (${K}_{\theta }$) is equal to 100 (Kcal/mol)/θ^2^ for different angles, where θ_0_ represents the equilibrium angle. The energy was minimized using the conjugate gradient method.

After that, Newton’s second law of the equation is utilized as the gradient of the potential function to describe the evolvement of particles as a function of time.

13 $${\text{F}}_{{\text{i}}} = \sum\nolimits_{{{\text{i}} \ne {\text{j}}}} {{\text{F}}_{{{\text{ij}}}} = {\text{m}}_{{\text{i}}} } \frac{{{\text{d}}^{2} {\text{r}}_{{\text{i}}} }}{{{\text{dt}}^{2} }} = {\text{m}}_{{\text{i}}} \frac{{{\text{dv}}_{{\text{i}}} }}{{{\text{dt}}}}$$

14 $${\text{F}}_{{{\text{ij}}}} = - {\text{grad}}\,{\text{V}}_{{{\text{ij}}}}$$

where ${r}_{i}$ is the position of atom i, m_i_ is atomic mass, V_ij_ is the potential function, and dt is MD time step. It is worth mentioning that the Velocity-Verlet algorithm owing to its stability and simplicity, fulfills motion equations’ association, in common in MD simulations.

In Eq. (9), the momentum $({{\text{P}}}_{\mathfrak{i}}$) can be described as below:

15 $${\text{P}}_{{\text{i}}} = {\text{ m}}_{{\text{i}}} {\text{v}}_{{\text{i}}}$$

The total energy (E) is representable through the Hamiltonian of atomic structures. When considering N atoms, it can be formulated as follows:

16 $${\text{H}}\left( {{\text{r}},{\text{ p}}} \right) \, = { 1}/{2}\sum\nolimits_{i} P + {\text{ U }}(r_{1 } + r_{2} ) = {\text{E}}$$

The total forces acting on each atom are calculated using the potential function, outlined as follows:

17 $$\frac{dU}{{dr_{i} }} = - m_{i} a_{i} = - F_{i}$$

In these simulations, the equations of motion can be solved using the Verlet algorithm. This computational method is described as follows^129^:

$$\begin{gathered} {\text{r }}({\text{t }} + \Delta t) = {\text{r}}\left( {\text{t}} \right) + {\text{v}}\left( {\text{t}} \right) + \Delta t + {1}/{\text{2 a}}\left( {\text{t}} \right) \, \Delta {\text{t}}^{{2}} + {\text{O}}\Delta {\text{t}}^{{4}} \hfill \\ {\text{v }}({\text{t }} + \Delta t) = {\text{ v}}\left( {\text{t}} \right) \, + {\text{a}}\left( {\text{t}} \right) \, + {\text{ a}}({\text{t }} + \Delta t)^{2} + \Delta t + {\text{O}}\Delta {\text{t}}^{2} \hfill \\ \end{gathered}$$

Furthermore, the Gaussian distribution employed for estimating the temperature of atoms can be expressed using the equation below:

18 $$3/2K_{B} {\text{T}} = {1}/{\text{N}}_{{{\text{atom}}}} \sum\nolimits_{i = 1}^{N} {\frac{1}{2} mv_{i}^{2} }$$

Following energy minimization, in this step, the Nose–Hoover thermostat is used to stabilize the temperature of the atomic system at T = 300 K as the starting conditions, using a time step of 1 fs, to eliminate any hot spots in the initial geometry, the NVT ensemble is used. Notably, the migration of AMIBs to the active sites of the anode material is facilitated by the electric field effect. Consequently, an external electric field of approximately 1.5 Vnm^−1^ is applied in the current study to improve system performance.

The Particle–Particle Particle-Mesh (PPPM) method^130^ is applied to the behavior of long-range electrostatic interactions. During the simulation, all systems are simulated in a canonical ensemble; and the nano-anode materials is equilibrated for 10 ns.

## Result and discussion

### MD simulation

Molecular dynamics (MD) simulation is a useful tool to study diffusion processes in battery electrode materials, and the LAMMPS is a powerful MD simulation developed^131^. LAMMPS is a molecular dynamics code designed for parallel platforms, with a focus on efficient execution, particularly in handling long-range coulomb interactions and developed by Plimpton et al.^132^. In this regard, MD simulations for anode material are performed using the LAMMPS program, which hosts 30 alkaline ions (li^+^/Na^+^ and K^+^). The LAMMPS MD code has been carried out to understand the ability of the 2D carbon-coated anode nanosheets for superior LIBs, SIBs, and PIBs storage. The high pseudo-capacitive contribution of the prevention of self-aggregation in MoSe_2_/MXene with a carbon layer is primarily attributed to its two-dimensional structure. Additionally, it offers a substantial surface area that effectively facilitates the adsorption and desorption of alkali ions (K^+^/Na^+^/Li^+^) throughout the charge and discharge cycles.

**Figure 2:**
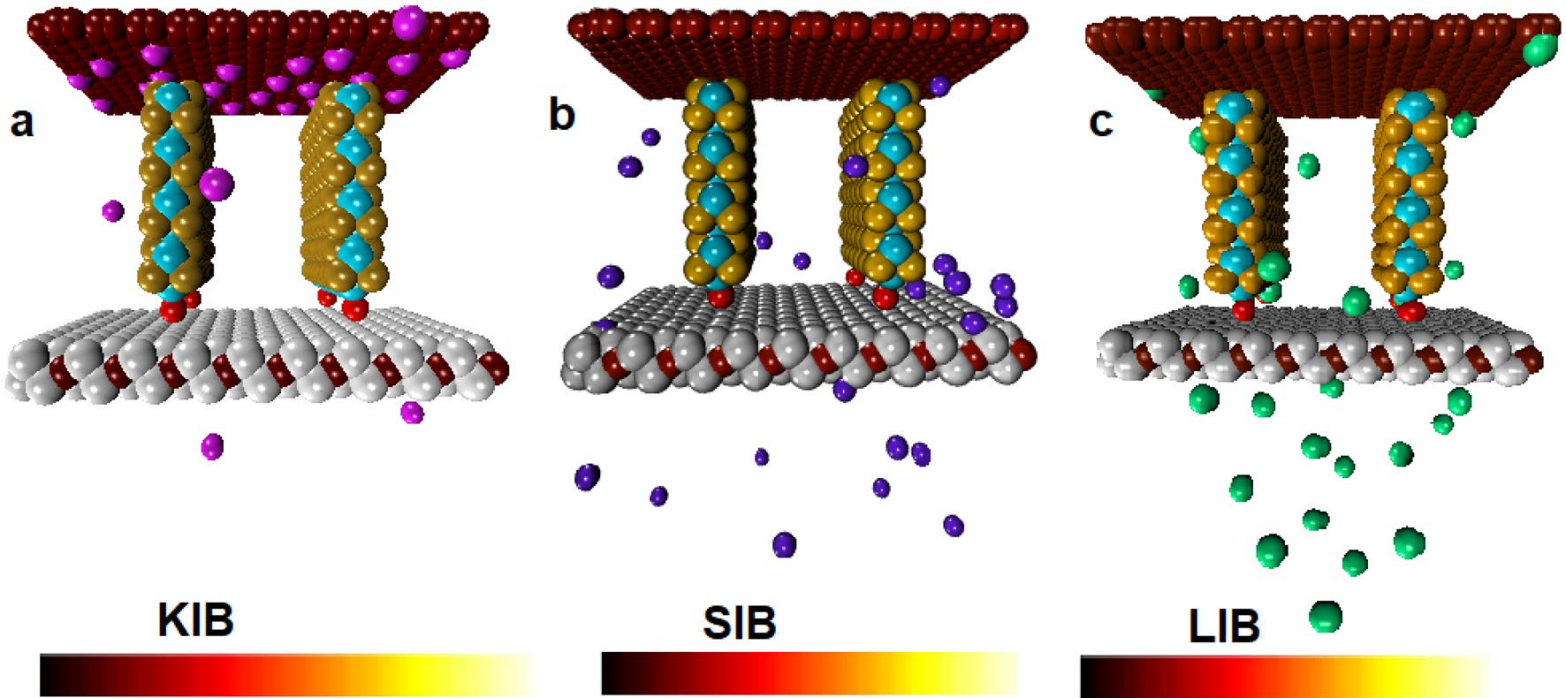
The snapshots of the final anode structures. (**a**) PIBs system (**b**) SIBs system (**c**) LIBs systems.

Our simulations have been divided into three setups (see Table 1): (a) MoSe_2_/MXene@C for LIBs; (b) MoSe_2_/MXene@C for SIBs; (c) MoSe_2_/MXene@C for PIBs. The initial and final snapshots of the molecular structures of the LIBs/SIBs/PIBs models during 10 ns MD simulations are shown in Figs. 1 and 2. It’s worth noting that the distance between the carbon layer cover and the MoSe_2_ nanosheets after full optimization for anode material design is about 6.42 Å. Additionally, the distance between each layer of the MoSe_2_ nanosheets is roughly 20 Å. The distance between the AMIBs and Mexene is approximately ~ 20 Å, and the interatomic distance varies from 4 to 5.5 Å. The great capability and stability of energy storage of the MoSe_2_@MXene via carbon layer as anode could be attributed to a hybrid nanostructure by rational design and coupling with conductive electrodes. It’s important to note that the volume variation occurring at the anode while loading ions is crucial. Excessive changes in volume can potentially harm the battery’s structure and raise safety issues.

As seen in Fig. 2, less swelling occurred for the anode systems because Li^+^, Na^+^ and K^+^ are preferentially migrated in the surface regions of the anode material, giving it a higher density. Overall, most SIBs and LIBs migrate around the surface of the MXene flakes and MoSe_2_ nanosheets interface. On the other hand, the difference in the adsorption of PIBs between the pores of anode material and the top or inner location of the anode is greater than that of Na^+^ and Li^+^, indicating a higher preferential tendency to migrate toward the carbon layer.

**Figure 3:**
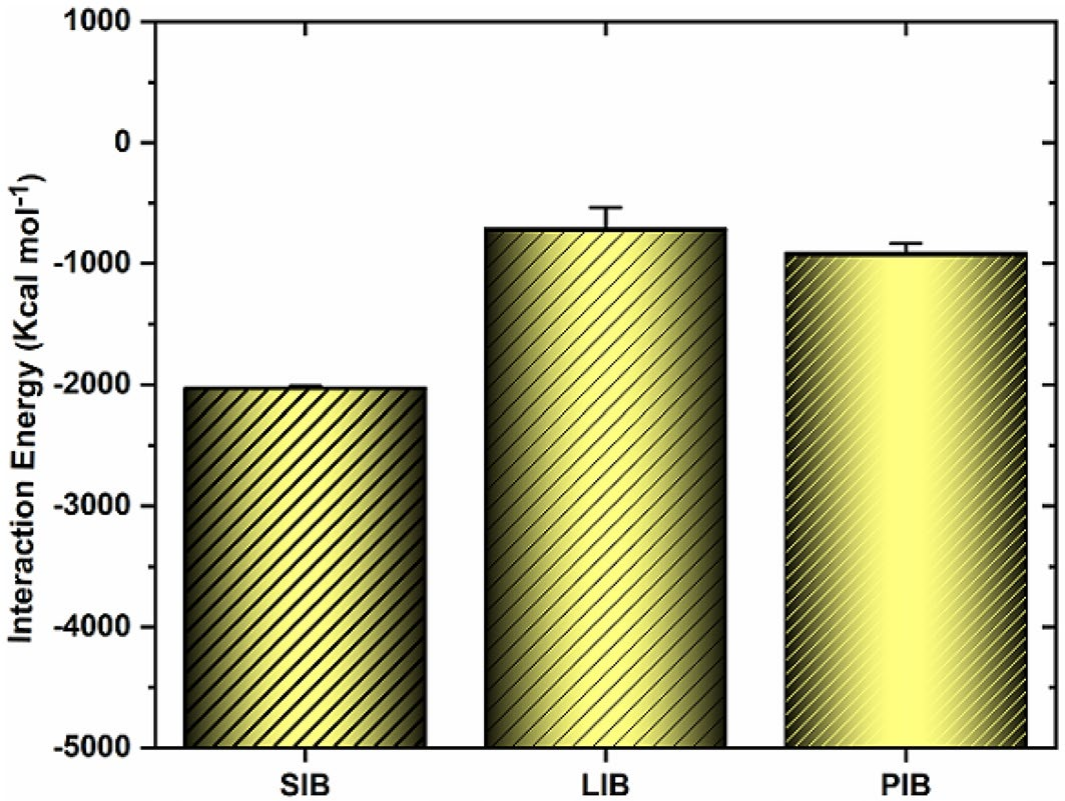
The potential energy of the studied systems. For the SIBs, PIBs and LIBs anode. The error bars represent the standard deviations of the data.

Meanwhile, because of the ionic size difference (Li^+^: 0.76 Å, Na^+^: 0.97 Å, K^+^: 1.38 Å), it leads to a decrease in diffusion and an increase in the penetration of LIBs compared to SIBs or PIBs during charge/discharge. Thus, pores’ shape and size directly influence alkali ion migration to 2D nanostructure. However, the anions tend to move towards the anode, marking the practical end of the charge cycle at 10 ns. The average of the potential energy of alkaline ions with anode material show varying negative values in the following sequence: system-SIB > system-PIB > system-LIB (are about ~ − 2030/41 > − 912/36 > − 713.65 kcal mol^−1^). And also, the interaction energies of the studied systems is illustrated in Fig. 3. As seen, the potential energy of Na^+^ is more negative than that of Li^+^ and K^+^, which seems Na^+^ to be significantly interacting with the surface of the anode material; this behavior may be related to the mobility and size of the ions. Following the migration process of LIBs/SIBs/PIBs toward the anode material, it is observed that the energy of Li^+^ is lower than that of other ions, which indicates that Li^+^ are more mobile than that Na^+^ and K^+^. Worthwhile to be noted that the MXene has high electrical conductivity and suitable intracavity spacing for LIBs. So Li^+^, during the charge/discharge process, can easily move inner or outside the anode material without any barriers. Therefore, due to the smaller size/weight and lower mass of the Li^+^, the LIBs-system allocated less energy than other systems. Indeed, compared to Li^+^, Na^+^ and K^+^ have larger ionic radii and, thus, lower charge density. A larger radius indicates less mobility and less ion transport; thus, K^+^ is strongly adsorbed to nano-anode materials compared to Li^+^ and Na^+^. This result can be ascribed to the distinctive feature of MoSe_2_/MXene@C, along with the excellent mobility of Na^+^. Notably, this unique anode not only offers well-prepared storage locations for Na^+^ than Li^+^/K^+^, but also diminishes local current density, thereby governing the deposition behavior of Na^+^^133^. The central part of our composite material, MoSe_2_/Mo_2_CTx/C, contained of Se–Mo–Se layers. Notwithstanding that, the Se and Mo atoms formed via covalent bonds within each layer, the layers with themselves held together through weak vdW forces. This layered structure was particularly favorable for the entry of Na^+^ into the MoSe_2_ interlayer space. Furthermore, due to the 2D nanostructure of MoSe_2_/MXene@C electrode, the reduced Na^+^ diffusion pathway and the ample storage sites have been supplied. Besides, the experimental work of Huang et al.^111^, presented the Carbon-Coated MXene@MoSe_2_ as a material for storing K^+^. Their results identified with an outstanding rate performance with 183 mA h g^−1^ at 10.0 A g^−1^ and a high reversible capacity of 355 mA h g^−1^ at 200 mA g^−1^ after 100 cycles. Beside, Yin and co-workers^115^ innovatively crafted a sandwich-structured MoSe_2_/MXene film to serve as an anode for Na^+^/Li^+^ capacitors. The electrode design, characterized by the sandwich structure in MoSe_2_/MXene, exhibits a notably elevated after 100 cycles discharge capacity of 281 mAh g^−1^.

### Root mean square deviation

The stability of the artificial anode material for the studied systems, such as LIBs/SIBs/PIBs, can be investigated by viewing the time dependence root mean square deviation (RMSD). This study serves dual purposes: validating the accuracy of our design and ensuring that our systems are in a state of conformational equilibrium. The difference between the initial and the final configuration is measured by the root mean square deviation (RMSD), presented by Eq. (6)^134^:

19 $${\text{RMSD}} = \sqrt { {{\sum\nolimits_{i = 1}^{N} {m\left( {r_{i} - r_{ref} } \right)^{2} } } \mathord{\left/ {\vphantom {{\sum\nolimits_{i = 1}^{N} {m\left( {r_{i} - r_{ref} } \right)^{2} } } {\sum\nolimits_{i = 1}^{N} {m_{i} } }}} \right. \kern-0pt} {\sum\nolimits_{i = 1}^{N} {m_{i} } }}}$$

where r_i_ (r_i,x_, r_i,y_, r_i,z_) is the coordinates of atom i at a certain instance, r_ref_ (r_ref,x_, r_ref,y_, r_ref,z_) corresponds to the coordinates of the atom i at its reference position and m_i_ is the mass of atom i.

**Figure 4:**
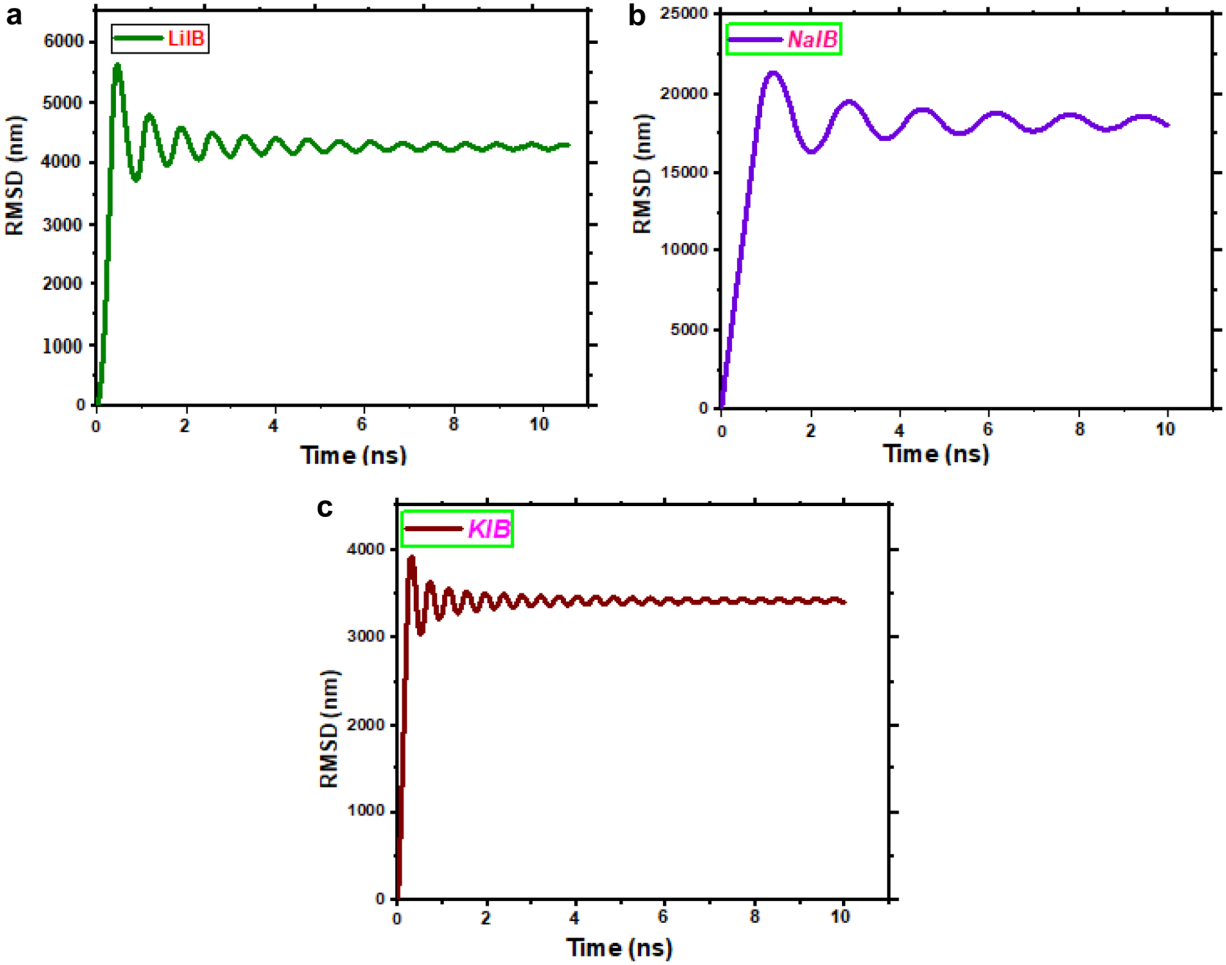
The RMSD for three different anode systems. (**a**) LIBs system (**b**) SIBs system (**c**) PIBs system.

The RMSD curves of simulated trajectories of different alkali ions of Li^+^, Na^+^, and K^+^ toward the nano-anode in the 20 ns, are evaluated in Fig. 4.

It is seen that the RMSD curves for all systems exhibit regular geometric fluctuations. As shown in Fig. 4, after 2 ns, the morphology of MoSe_2_/MXene@C systems well maintained stable until the end of the simulation time, at 20 ns. It is worth mentioning that the anode material structure provides a short path with a large contact surface for the transfer/diffusion of Na^+^, K^+^ and Li^+^, which leads to improved cycle stability and speed performance. For instance, Huang et al. designed and constructed 3D architectures of a few-layered Ti_3_C_2_/NiCo_2_Se_4_ as anode material for SIBs through the solvothermal method^135^. Interestingly, this cycle stability has also good agreement with experimental works. Yin’s group^115^ engineered a flexible MoSe_2_/MXene electrode, serving as an anode for both SIBs and LIBs, to achieve a robust cycle performance. The MoSe_2_/MXene sandwich structure, observed during the insertion and extraction of Na^+^/Li^+^, demonstrates the capability to achieve high energy and power densities, reaching maximum values of 110.1 Wh kg^−1^ and 4764.7 W kg^−1^, as well as 84.9 Wh kg^−1^ and 3288.3 W kg^−1^, respectively.

### Ion diffusion

The mean-square displacement (MSD) is investigated to study the migration behaviour of LIBs/SIBs/PIBs toward the anode material.

The mean squared displacement (MSD) over time is employed to evaluate how target particle(s) diffuse within a system and can provide a good approximation of diffusion which is defined as:

20 $${\text{MSD }}\left( {\text{t}} \right) \, = {\raise0.7ex\hbox{$1$} \!\mathord{\left/ {\vphantom {1 N}}\right.\kern-0pt} \!\lower0.7ex\hbox{$N$}}\sum\nolimits_{i = 1}^{N} {\left\langle {\left[ {r_{i} \left( {t_{0} + t} \right)^{2} } \right] - \left[ {r_{i} (t_{0} )} \right]^{2} } \right\rangle }$$

where r_i_ (t) describes the position of i atoms at time t, r_i_ (t_0_) is the original position of i atoms, N denotes the number of atoms, and the angular bracket < > denotes the time average.

**Figure 5:**
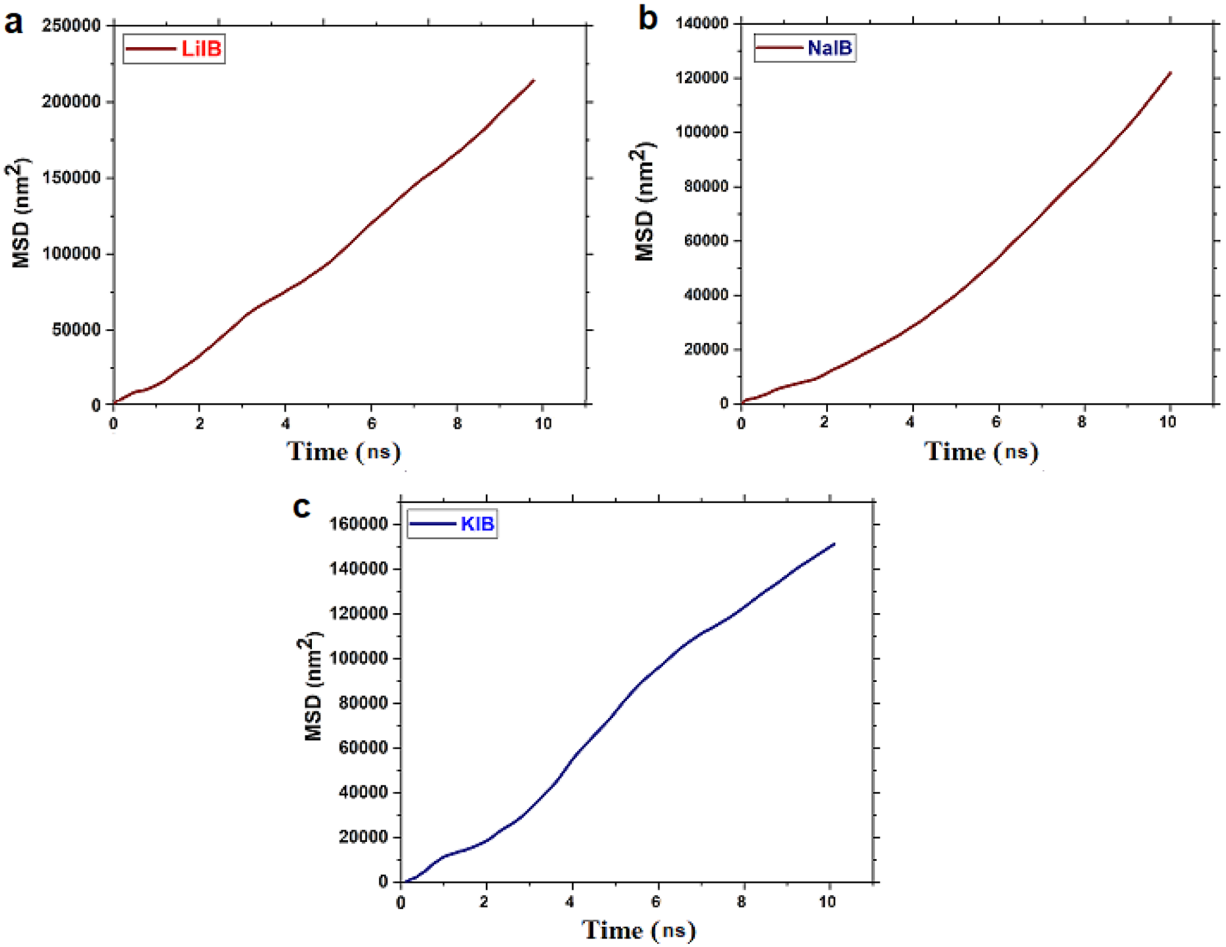
MSD patterns for investigated systems. (**a**) LIBs system (**b**) SIBs system (**c**) PIBs system.

We plotted the MSD versus the time to ensure enough time had elapsed for the simulations. Furthermore, the MSD curves of each system indicate that the ions in the different systems have different diffusion tendencies. The highest MSD value at time t, indicating that the ions quickly diffuse and are far away from their initial positions. Figure 5 illustrates the MSD curves for (a) Li^+^, (b) Na^+^ (c) K^+^ along the z-axis toward the anode material in the simulated systems. As depicted in Fig. 5, the MSD curve is linear, and its slope enhances with time. This finding confirms that the long-range AMIBs migrate toward the corresponding anode material structure. However, the slope of the MSD curve for Li^+^ is more than the other ions; thus, it can be concluded that the migration of Li^+^ is less than two other ions. This phenomenon can be attributed to the weaker diffusion of Li^+^ than to Na^+^/k^+^. In addition, this behaviour is caused by the repulsive interactions between monovalent Li^+^ and anode material, which is due to the small size of Li^+^ and their higher charge density. So, in the LIBs system, the proposed anode cannot be suitable for the storage of LIBs and has no excellent cycling ability. In contrast, the Na^+^ are allowed to migrate almost freely along the direction of the anode materials; furthermore, they prefer to coordinate into the internal space of the anode active materials. Several studies have reported on SIBs storage; for instance, Xie et al.^136^ reported the porous Ti_3_C_2_Tx (p-Ti_3_C_2_Tx) MXene as a new electrode material for high-rate Na^+^ storage. Their XRD findings illustrate that the porous configuration of p-Ti_3_C_2_Tx effectively minimizes the distance for electrolyte movement, promoting swift transport and diffusion of Na^+^ throughout the charging/discharging cycles. Even when subjected to increased current densities of 1 and 10 A g^−1^, the p-Ti_3_C_2_Tx electrodes consistently preserved capacities of 166 Ma h g^−1^ and 124 mA h g^−1^, respectively. Notably, even at an extremely high current density of 100 A g^−1^, the electrodes showcased a capacity of 24 mA h g^−1^ with an outstanding coulombic efficiency, achieving an impressive high-rate capability of 99%.

Their XRD results demonstrate that the porous structure of p-Ti_3_C_2_Tx significantly reduces the distance that electrolytes to facilitate the rapid transportation and diffusion of Na^+^ during the charging/discharging cycle. At high current densities of 1 and 10 A g^−1^, the p-Ti_3_C_2_Tx electrodes consistently retained capacities of 166 mA h g^−1^ and 124 mA h g^−1^, respectively. Remarkably, even at an exceptionally high current density of 100 A g^−1^, the electrodes demonstrated a capacity of 24 mA h g^−1^ with a remarkable coulombic efficiency, reaching a high-rate capability of 99%.

Besides, MXenes has been reported that MXenes can be used as electrode materials for storing SIBs^137–139^. Moreover, the unique attributes of the MoSe_2_/MXene@carbon-coated nanosheet can be ascribed to the exceptionally conductive functionalized MXene substrate, specifically the (Ti–O–Mo) nanorods. These nanorods contribute to a shortened Na^+^ diffusion distance and provide abundant storage sites, enhancing overall performance. Besides, MoSe_2_/MXene@C acknowledged as a superior anode material, can proficiently diminish the Na^+^ diffusion barrier and reveal a greater number of active sites for Na^+^ adsorption^133^. In particular, the anchoring of 2D bimetal nanosheets on the MXene substrate offers a high specific capacity and mitigates volume expansion. Moreover, the MXene substrate serves to shorten the diffusion pathway of Na^+^ and enhance electronic conductivity. Meanwhile, PIBs tend to adsorb onto the surface of the graphene, so they have less mobility during the charge/discharge process. Therefore, K^+^ adsorbing by abrupt jumps on the carbon layer surface would form potassium clusters limiting graphene’s application as an anode material. However, this evidence shows that Na^+^ are much more active than K^+^, so Na^+^ diffuse much faster than K^+^ toward the anode materials during the charging and discharging process. Since the frequency of oscillations in the MSD curves of AMIBs is low, it can be assumed that the systems during the migration of ions toward the anode material reach convergence and a stable state. Specifically, the hierarchical porous structure based on nanosheets with ultrathin 2D architecture transport path for electron and alkali ions diffusion. And also provides a larger contact area between anode and ions, which leads to improved charge transfer resistance during cycling and speed performance. Nevertheless, the fast transport of ions can be attributed to the excellent performance of the MoSe_2_/MXene@C structure, mainly due to the carbon coating and its surface interactions.

Based on the findings, Na^+^ release exhibited a good tendency toward the anode materials, with a high capacity during simulation. This observation aligns well with the experimental results. As example, Tan’s research group^140^ investigated a new material called MoSe_2_@Mo_2_CTX/C, which has great ionic conductivity and maintains its structural integrity during the insertion and release of sodium ions. They introduced this substance into a half-cell and examined it under rates of 0.5, 1, and 2 A g^−1^ for 2200 cycles. The obtained capacities stood at 652.2, 484.4, and 238.4 mAh g^−1^, while the capacity retention values were associated about 102.5%, 103.9%, and 86.3%. Their findings represented that MoSe_2_@Mo_2_CTX/C has good potential as an anode material for sodium-ion batteries. On the other hand, Wang et al.^141^ reported that both theoretical and experimental assessment of layered MoSe_2_ nanoplates as the anode materials. The results illustrated well potential of MoSe_2_ as an anode material for SIBs. They discovered that the theoretical specific capacity of bulk MoSe_2_ can reach up to 422.28 mAh g^−1^. Additionally, their XRD findings revealed that MoSe_2_ exhibits impressive initial discharge/charge capacities of 513 and 440 mAh g^−1^, along with excellent cycling performance.

#### Computation of pair distribution functions

The radial distribution function (RDF) generally describes the probability of finding a particle at a distance r from the reference particle^142^. RDF analysis can explain the migration of AMIBs toward anode material, which provides significant insight into the accumulation of alkali ions formed around LIB/SIB/PIB systems. The radial distribution function for a pair of species ij is denoted as gij(r) and the mentioned relationship is mathematically represented by the following equation:

21 $${\text{g}}_{{{\text{ij}}}} ({\text{r}}) = {\raise0.7ex\hbox{${n\left( r \right)}$} \!\mathord{\left/ {\vphantom {{n\left( r \right)} {4\pi r^{2} dr\rho }}}\right.\kern-0pt} \!\lower0.7ex\hbox{${4\pi r^{2} dr\rho }$}}$$

**Figure 6:**
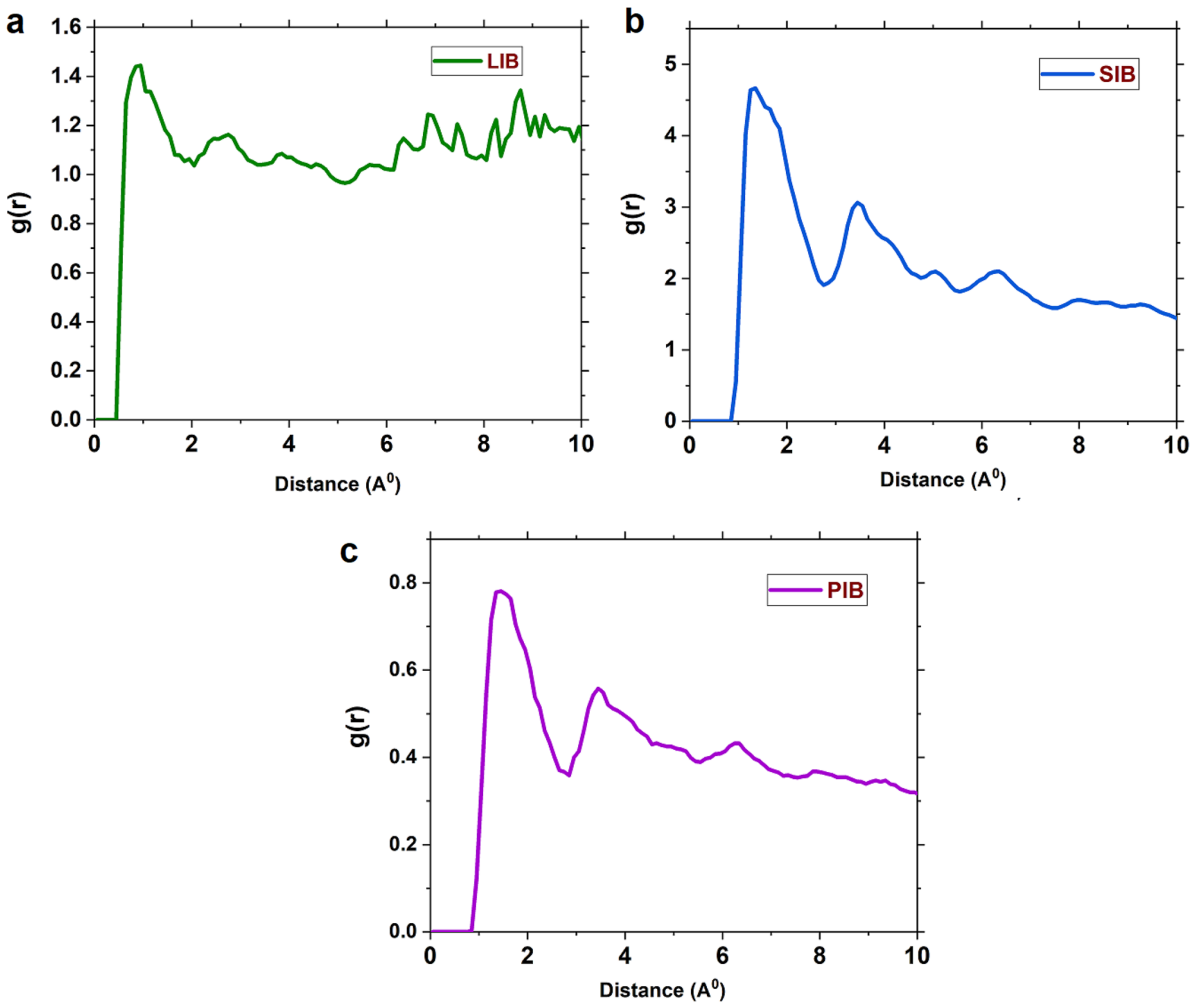
RDFs of the three alkali ions with respect to the anode materials surface in the investigat-ed systems. (**a**) LIBs system (**b**) SIBs system (**c**) PIBs system.

The expression describes the quantity n(r), which represents the number of j ions or molecules found at a radial distance r from the location of the i ion or molecule. Here, 4πr^2^ dr represents the volume of a shell with a thickness of dr at the radial distance r, and ρ is the bulk number density of the j molecule or ion. The results of RDF analysis for the alkali ions are illustrated in Fig. 6. It can be observed from this Figure RDF is zero for a short distance and is not observed any sharp peak in this region, owing to strong repulsive forces between AMIBs and anode material in the short range (> 0.4 nm). The RDF diagram shows distinct peaks in the systems containing LIBs/SIBs/PIBs; the order of their peak heights is SIBs-system > PIBs-system > LIBs-system. As seen in Fig. 6, the LIBs system’s RDF peak is weak intensity due to the weak interaction of Li^+^ with anode materials and which appeared at about ~ 1.45 nm. On the other hand, in the LIBs system, some Na^+^ are trapped by Mexen, interacting with existing titanium atoms, preventing the passage of other Li^+^ towards the anode material. The obtained results showed that the highest peak of the RDF belongs to the Na^+^ in the SIB anode system, which appeared at about ~ 0.85. Notably, it can be ascribed to Mo–O–Ti bonds, indicating the creation of covalent bonds at the interface MXene flakes and, MoSe_2_ nanosheets, significantly enhancing the interaction of the two components. Moreover, the exceptional adsorption capacity of SIBs is a direct result of their profound affinity for the material’s surface, effectively impeding the migration of Na^+^, where the coulombic interaction between the anode materials and monovalent alkaline ions is much greater than the other ions.

Research groups have theorized that Na^+^ can diffuse via low-dispersion boundary layers, which makes MXenes effective for SIB applications. This property is useful for constructing MXene-based SIB anodes^114–120^. It is worth noting that these results agree with the results of the previous sections. A close inspection of Fig. 6 indicates that in the PIBs system, a strong peak appeared at around 1.35; it confirms that the resident time of ions on their respective sites is much longer than their spent time outside of the hopping locations site. This fact can be attributed to the radius of PIBs; thus, large ions of K^+^ so strongly adsorbed on the active sites at the PIBs anode surface. Therefore, indicating that the time spent in the active site of anode material is significantly longer than the hopping time. In addition, the large radius of K^+^ makes anode materials challenging during repeated K^+^ insertion/extraction. Yuan et al.^35^ devised a novel approach by designing highly conductive 3D hierarchical network composites, NiSe_2_@C@MXene, for advanced sodium ion storage. The synergistic combination of these components resulted in exceptional reversibility and long-term stability, demonstrated by a capacity of 327 mA h g^−1^ at 2000 mA g^−1^ after 4000 cycles.

In summary, the exceptionally conductive MXene substrate can improve electronic conductivity by anchoring nanosheets (MoSe_2_) chemical groups. Additionaly, the carbon layer strengthens the composite structure and further increases the hybrid nanosheets’ overall conductivity. Most importantly, the key point is achieving fast LIBs/SIBs/PIBs diffusion kinetics in the MoSe_2_/MXene@C interlayer. In this regard, this study emphasized that in the SIB/PIB anode systems, the presences of Na^+^ and K^+^ are more likely than Li^+^ around the anode material.

We found that the presence of MXene helped in shortening the path of Na^+^ diffusion and prevented the degradation of MoSe_2_ nanosheets due to their large volume expansion that occurs during its interaction via the Na^+^ and electrode. Furthermore, the morphology of the material remained unchanged after simulation, indicating that it has a certain level of stability. Finally, it is found that Na^+^ has a more negative potential energy compared to Li^+^ and K^+^. This indicates a significant interaction of Na^+^ with the anode material surface. This phenomenon occurs due to the weaker diffusion of Li^+^ compared to Na^+^/K^+^. The repulsive interactions between the anode material and monovalent Li^+^ are the primary reason behind this behavior due to the small size of Li^+^ and its higher charge density. It’s important to note that the findings validate the idea that the systematic nanostructure design of carbon-coated MoSe_2_-MXene has the potential to greatly improve the storage performance of AMIBs. The hierarchical anode materials demonstrated great performance in the aspect of elevated capacity and exceptional rate capability for advanced monovalent ion batteries (AMIBs), particularly in the case of SIBs. Notably, 2D highly conductive MXene serves as a suitable SIBs host^119^ and enhances reaction kinetics when coupled with transition metal oxides (TMOs) (such as MoS_2_ and MoSe_2_)^104^.

Nevertheless, the fast transport of ions can be attributed to the excellent performance of the MoSe_2_/MXene@C structure, mainly due to the carbon coating and its surface interactions.

## Data Availability

Authors can confirm that all relevant data are included in the article and/or its supplementary information files.

## Code availability

The codes that support the findings of this study are available from the corresponding authors upon reasonable request.
